# New insights into the immunopathology of early *Toxocara canis* infection in mice

**DOI:** 10.1186/s13071-015-0962-7

**Published:** 2015-07-02

**Authors:** Nathália M. Resende, Pedro Henrique Gazzinelli-Guimarães, Fernando S. Barbosa, Luciana M. Oliveira, Denise S. Nogueira, Ana Clara Gazzinelli-Guimarães, Marco Túlio P. Gonçalves, Chiara C. O. Amorim, Fabrício M. S. Oliveira, Marcelo V. Caliari, Milene A. Rachid, Gustavo T. Volpato, Lilian L. Bueno, Stefan M. Geiger, Ricardo T. Fujiwara

**Affiliations:** Departamento de Parasitologia, Instituto de Ciências Biológicas, Universidade Federal de Minas Gerais, Belo Horizonte, Brazil; Laboratório de Fisiologia de Sistemas e Toxicologia Reprodutiva, Instituto de Ciências Biológicas e da Saúde, Universidade Federal de Mato Grosso, Barra do Garças, Brazil; Departamento de Patologia, Instituto de Ciências Biológicas, Universidade Federal de Minas Gerais, Belo Horizonte, Brazil

**Keywords:** *Toxocara canis*, Immunopathology, Murine model, Cytokines, Inflammation

## Abstract

**Background:**

Nematodes of the genus *Toxocara* are cosmopolitan roundworms frequently found in dogs and cats. *Toxocara* spp. can accidentally infect humans and cause a zoonosis called human toxocariasis, which is characterized by visceral, ocular or cerebral migration of larval stages of the parasite, without completing its life cycle. In general, chronic nematode infections induce a polarized T_H_2 immune response. However, during the initial phase of infection, a strong pro-inflammatory response is part of the immunological profile and might determine the outcome and/or pathology of the infection.

**Methods:**

Parasitological aspects and histopathology during larval migration were evaluated after early *T. canis* experimental infection of BALB/c mice, which were inoculated via the intra-gastric route with a single dose of 1000 fully embryonated eggs. Innate immune responses and systemic cytokine patterns (T_H_1, T_H_2, T_H_17 and regulatory cytokines) were determined at different times after experimental challenge by sandwich ELISA.

**Results:**

We found that experimental infection with *T. canis* induced a mix of innate inflammatory/T_H_17/T_H_2 responses during early infection, with a predominance of the latter. The T_H_2 response was evidenced by significant increases in cytokines such as IL-4, IL-5, IL-13 and IL-33, in addition to increasing levels of IL-6 and IL-17. No significant increases were observed for IL-10, TNF-α or IFN-γ levels. In parallel, parasitological analysis clearly revealed the pattern of larval migration through the mouse organs, starting from the liver in the first 24 h of infection, reaching the peak in the lungs on the 3rd day of infection and finally being found numerously in the brain after 5 days of infection. Peripheral leukocytosis, characterized by early neutrophilia and subsequent eosinophilia, was remarkable during early infection. The tissue damage induced by larvae was evidenced by histopathological analysis of the organs at different time points of infection. In all of the affected organs, larval migration induced intense inflammatory infiltrate and hemorrhage.

**Conclusion:**

In conclusion, these new insights into early *T. canis* infection in mice presented here enabled a better understanding of the immunopathological events that might also occur during human toxocariasis, thus contributing to future strategies of diagnosis and control.

## Background

Toxocariasis is caused by ingestion of fully embryonated eggs or ingestion of infective larvae together with paratenic hosts of the nematode roundworms *Toxocara canis* (Werner 1782) or *Toxocara cati* (Schrank 1788), which are parasites of dogs and cats, respectively. These species are widespread and, as a zoonotic infection, can be transmitted to humans. Human toxocariasis, characterized by visceral larval migration, is considered an important public health problem in the tropics and in sub-tropical nations where pet treatment and population control are limited [1]. This scenario is aggravated by precarious sanitation in these environments or by deficiencies in the diagnosis and anthelmintic treatment of pets, thus exposing humans to these parasites [2].

Due to the increasing number of dogs and cats being kept as pets worldwide, *T. canis* and *T. cati* infections are the main causes of human toxocariasis [3]. Oge et al. [4] reported that worldwide prevalence rates vary from 3 to 82 % for *T. canis* in dogs and from 8 to 91 % for *T. cati* in cats. The differential diagnosis between *T. canis* and *T. cati* infections by serological surveys remains challenging [5] due to the large number of common antigenic fractions. However, there is no difference in the zoonotic potential of both parasite species, and they showed comparable behavior in paratenic hosts [6].

Human toxocariasis can be asymptomatic or can show clinical syndromes caused by the migration of *Toxocara* spp. larvae through the bloodstream and internal organs, depending on the intensity of infection and the immune response status of the accidental or paratenic host [1]. Depending on the affected organ and the clinical symptoms, toxocariasis can be classified as visceral larva migrans (VLM) or visceral toxocariasis (VT) and ocular larva migrans (OLM) or ocular toxocariasis (OT), covert or common toxocariasis (CT) and neurotoxocariasis (NT) [7]. Several studies have characterized human toxocariasis as a chronic infection that can persist for several years [8–10].

Helminth infection induces a polarized T_H_2 response, typically reflected in the secretion of IL-4, IL-5 and IL-13, cytokines that are involved in the activation of mast cells, eosinophils, and macrophages and the secretion of high levels of IgE. Triggering of the T_H_2 response normally coincides with downmodulation of the T_H_1 inflammatory response, reducing the expression of tumor necrosis factor alpha (TNF-α), interferon gamma (IFN-γ) and IL-17 [11, 12]. However, helminth parasites can evade the host immune system, e.g., by the activation of regulatory T cells that induce the production of downregulating cytokines, such as IL-10 and transforming growth factor beta (TGF-β) [13, 14]. Nevertheless, normally the immune system orchestrates a careful balance among pro- and anti-inflammatory and regulatory responses to function efficiently against *Toxocara* spp. infection, although the chronic survival of larvae in paratenic hosts indicates that this may not always be successful in eradicating all the worms.

In paratenic hosts, such as humans and mice, *T. canis* larvae do not develop to the adult stage but rather migrate throughout the somatic tissue and persist in the infectious L3 stage for extensive periods. In this context, experimental infection in mice, which mimics the biology of human infection, might be relevant to a better understanding of human toxocariasis [6, 8, 9]. Experimental *T. canis* infections in mice revealed elevated eosinophilia with high titers of IgE at 60 days after infection [15]. In addition, high plasma levels of IL-6 and IFN-γ were correlated with pulmonary lesions [16], and in chronic *T. canis* infections, a predominance of T_H_2 immune response was reported [17]. However, it remains unclear when and how the systemic T_H_1 response switches to the dominant T_H_2 immune response or whether these responses coexist. Thus, the purposes of this study were to evaluate the migratory route and histopathology of parasitized tissues and to correlate the results with innate immune responses and systemic cytokines profiles during early *T. canis* infection in mice.

## Methods

### Parasite

Adult *Toxocara canis* worms were obtained from the feces of naturally infected dogs, which were kept at the Zoonosis Control Center (Belo Horizonte, Minas Gerais, Brazil). At this center, the dogs were routinely treated with anthelmintics (Top-Dog, Ourofino®, Cravinhos, São Paulo, Brazil), and mature *T. canis* parasites were collected after elimination in fecal samples. The parasites were maintained in saline solution until processed at the Laboratory of Immunology and Genomics of Parasites at the Federal University of Minas Gerais, Brazil.

The purification of *T. canis* eggs was performed as described by Gazzinelli-Guimarães et al. [18]; i.e., the eggs were isolated from uteri via gentle mechanical maceration, and they were purified by straining and cultured to embryonation in 0.2 M H_2_S0_4_ solution. The eggs were incubated in 50 mL culture flasks and were kept in a controlled temperature chamber at 26 ± 1 °C, undergoing oxygenation three times per week by stirring. Embryonation was evaluated microscopically once per week, and the percentages of embryonated eggs were calculated by three independent experiments using three aliquots of 10 μL egg suspension for each time point.

### Animals

BALB/c mice, obtained from the Central Animal Facility of the Federal University of Minas Gerais, were kept on a normal light/dark cycle (12 h) in a climate-controlled environment (25 °C) throughout the study. The animals were maintained in collective cages and were fed animal food (Purina®) and provided with water ad libitum.

### Ethical approval

The mice were maintained and infected in accordance with institutional and national guidelines. The protocol was approved by the Ethics Committee for Animal Experimentation (CEUA) of the Federal University of Minas Gerais, Brazil (Protocol# 181/2013).

### Experimental infection

Experimental infection was performed using fully embryonated eggs from a single 6 week old culture. For inoculation, the culture of eggs was incubated with 5 % sodium hypochlorite in a CO_2_ incubator for 2 h to disrupt the outer egg layer, thus facilitating larval hatching, followed by five washes with saline solution to remove the sodium hypochlorite.

The animals were inoculated via the intra-gastric route by gavage, with a single dose of 1000 embryonated *T. canis* eggs in 0.2 mL of saline solution, followed by 0.1 mL of H_2_O to rinse the remaining eggs from the syringe and needle.

### Characterization of *T. canis* larval migration patterns in mice

BALB/c mice (8 weeks old, female) were euthanized at 1, 3, 5, 7 and 14 days post-infection (p.i.). The liver, lungs and brain were removed from six animals per time point of infection, and the organs were sliced finely with scissors and placed in a Baermann apparatus in PBS buffer for 4 h at 37 °C for total larval recovery. Subsequently, the larvae were recovered in the pellet, fixed with formalin (10 %) and quantified under a light microscope.

### Histopathology and morphometry of tissues of *T. canis*-infected mice

At 0, 7 and 14 days p.i., the liver, lungs and brain were removed from seven animals per time point of infection and were fixed in 10 % buffered formaldehyde at a pH of 7.2. After processing in alcohol and xylol, the fragments were embedded in paraffin, and 4 μm thick sections were obtained and stained with hematoxylin and eosin (H&E). The tissues were analyzed using KS400 software coupled to a Carl Zeiss image analyzer (Oberkochen, Germany), as previously described [19].

To evaluate tissue damage, the sections were thoroughly analyzed, and all of the digitized images of the liver and brain were captured at ×100 and ×400 magnification, respectively, using a JVC TK-1270/RGB micro-camera (Tokyo, Japan). All of the sections were digitized at 300 dpi resolution, and each image pixel was used to create a binary image to calculate the total area of tissue. The area, in square micrometers, of the lower section was used as a standard for the tissue for statistical analysis [20].

To evaluate the intensity of lung inflammation, the degree of septum thickening was calculated. Thirty random images were captured ×200 magnification, using a JVC TK-1270/RGB micro-camera (Tokyo, Japan), comprising an area of 13.2 × 10^6^ μm^2^ for statistical analysis [21].

### Systemic analysis

For hematological analysis, 36 mice at 0 (uninfected group), 1, 3, 5, 7 and 14 days p.i. were bled by conventional methods. The blood was then centrifuged, and the plasma was collected and frozen at −80 °C for further cytokine measurement.

### Hematological analysis

Global counts of erythrocytes, leukocytes and platelets were performed with a hematological analyzer (Bio 2900 Vet, Bioeasy, USA). For differential white blood cell counting, blood smears were Giemsa stained, and 100 white blood cells were differentiated under a light microscope.

### Cytokine profile

The profiles of plasmatic T_H_1, T_H_2, T_H_17 and regulatory cytokines were determined by quantification of IL-4 (DY404), IL-5 (DY405), IL-6 (DY406), IL-10 (DY417), IL-13 (DY413), IL-17 (DY421), IL-33 (DY3626), IFN-y (DY485) and TNF-α (DY410) by ELISA, according to the manufacturer’s instructions (R&D Systems, USA). The absorbance was determined by a VersaMax ELISA microplate reader (Molecular Devices, USA) at a wavelength of 492 nm, and the cytokine concentrations (pg/mL) for each sample were calculated by interpolation from a standard curve.

### Statistical analysis

Software GraphPad Prism software, version 6 (GraphPad Inc., USA), was used for the statistical analysis. To verify the data distribution, the D’Agostino-Pearson test and the Shapiro-Wilk normality test were used. Ordinary one-way ANOVA and Holm-Sidak’s multiple comparisons test were applied for the pattern of migration of *T. canis* larvae and for hematological profiles. Cytokine profiles were analyzed and compared with the Kruskal-Wallis test, followed by Dunn’s multiple comparisons test. The unpaired *t* test was used for the comparison of data from morphometric analysis. Statistically significant differences were considered when the *p* value was ≤0.05.

## Results

### In vitro *T. canis* egg embryonation over time

**Fig. 1:**
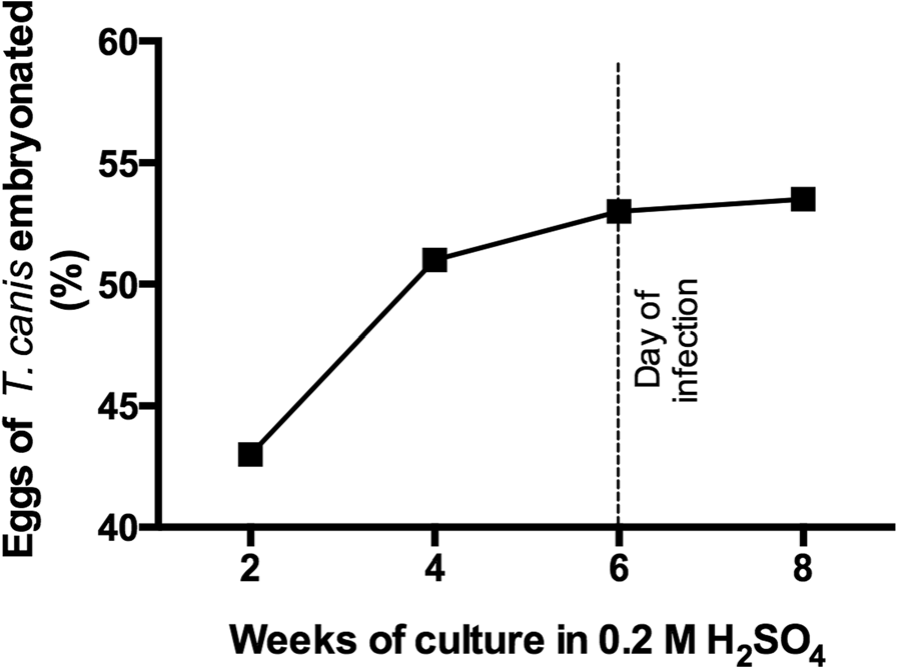
Embryonation index of *Toxocara canis* eggs

Approximately 700,000 eggs of *T. canis* were cultured in 0.2 M H_2_SO_4_, and the time required for egg embryonation was assessed over 8 weeks at 26 °C (Fig. 1). After 6 weeks of cultivation, 53 % of the eggs were fully embryonated, and the proportion of embryonated eggs remained stable until the end of the experiment.

### The pattern of migration of *T. canis* larvae in mice

**Fig. 2:**
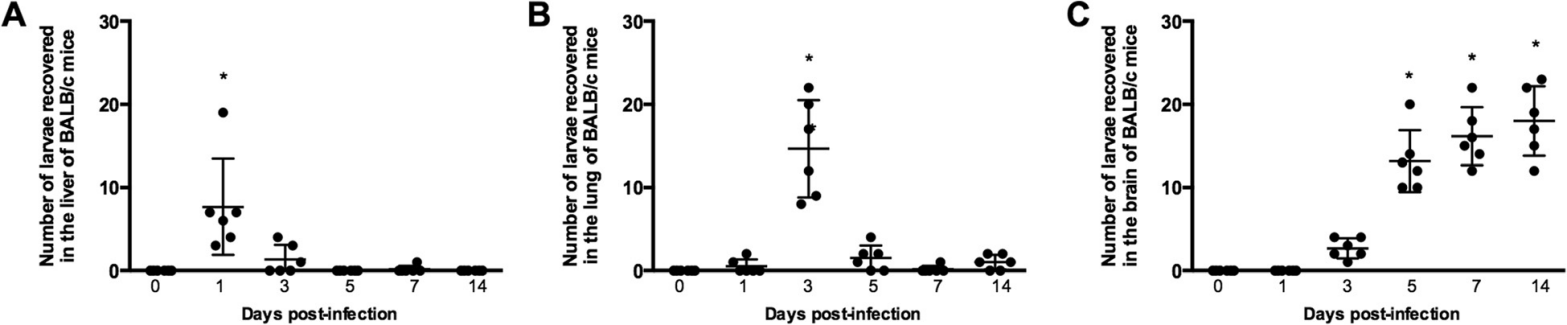
Migratory route of *Toxocara canis* larvae in BALB/c mice over 14 days of infection. **a** Number of larvae recovered from the liver. **b** Number of larvae recovered from the lungs. **c** Number of larvae recovered from the brain. Each group consisted of six animals per time point of infection. *Horizontal bars* represent mean values ± SDs. Significant differences between the groups are indicated by an *asterisk* (*p* ≤ 0.05)

To determine the pattern of migration of *T. canis* in early toxocariasis, larvae were recovered from the liver, lungs and brain at 0, 1, 3, 5, 7 and 14 days p.i. from infected BALB/c mice (Fig. 2). The results revealed that after 1 day p.i., *T. canis* larvae were predominantly recovered from the liver (Fig. 2a), while after 3 days p.i., the majority of larvae were found in the lungs (Fig. 2b). After this period of time, the larvae began to leave the lungs and migrate to the brain, where they were consistently found after 5 days p.i. and remained in situ at least for 14 days p.i. (Fig. 2c).

### Tissue damage caused by *T. canis* larva migration during early infection

To evaluate the damage caused by the migration of larvae into the tissues, the liver, lungs and brain were removed, and histopathological and morphometric analyses were performed under the light microscope.

**Fig. 3:**
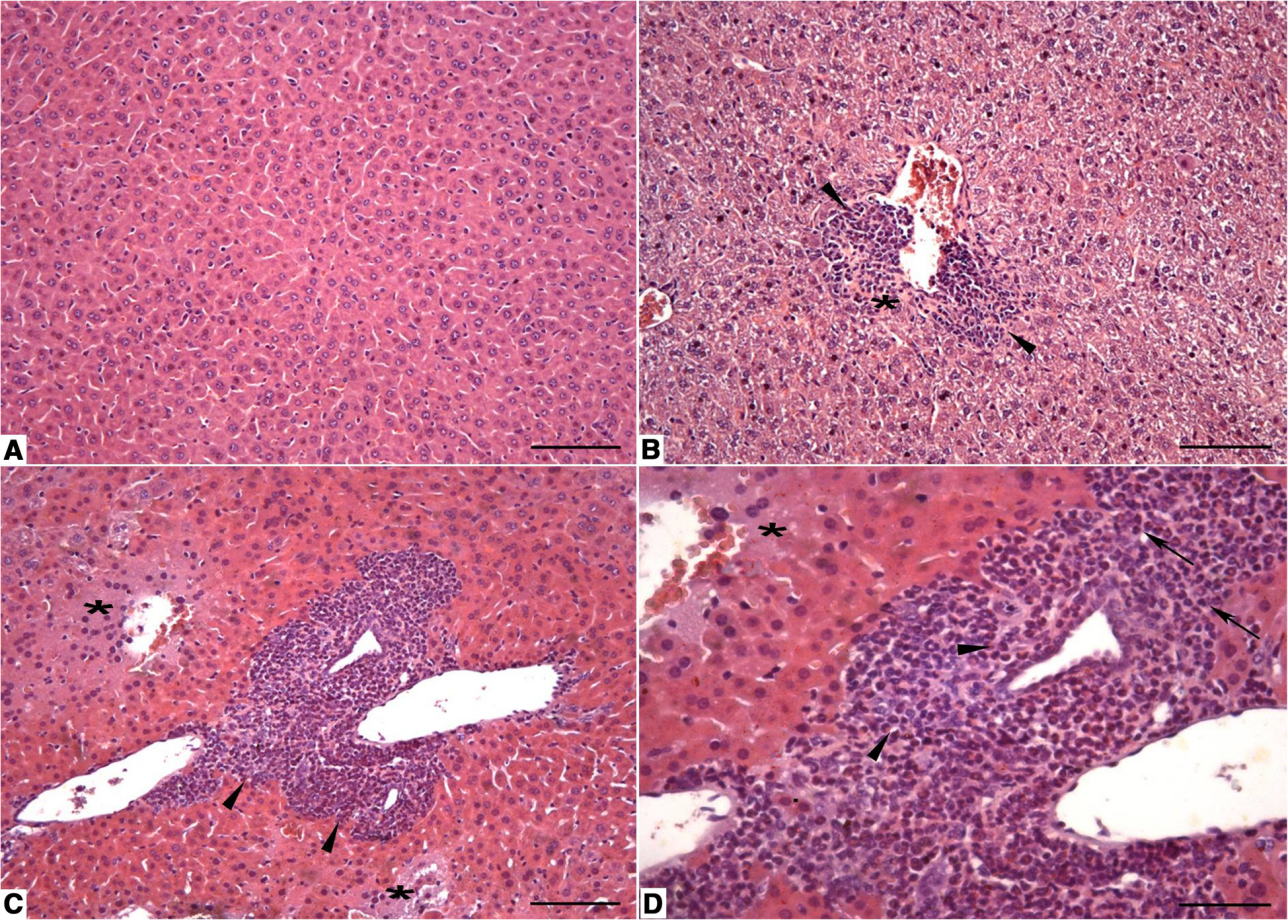
Liver parenchyma of *Toxocara canis*-infected BALB/c mice. **a** Control group: normal liver parenchyma with H&E staining. *Bar* = 100 μm. **b** 7 days post-infection (p.i.): inflammatory infiltration (*arrowheads*) and small area of hepatic necrosis (*). H&E. *Bar* = 100 μm. **c** 14 days p.i.: area with intense inflammatory infiltration (*arrowheads*) and hepatic necrosis (*). H&E. *Bar* = 100 μm. **d** 14 days p.i.: higher magnification of the previous figure showing inflammatory infiltrate consisting of eosinophils (*arrowheads*) and neutrophils (*arrows*), as well as necrosis (*). H&E. *Bar* = 50 μm

**Fig. 4:**
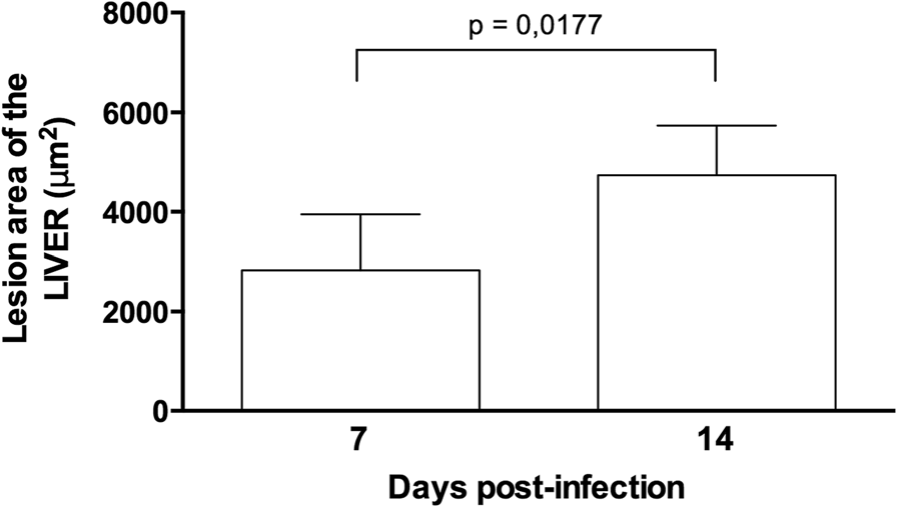
Morphometry of lesion areas in the livers of BALB/c mice at 7 and 14 days post-infection with *Toxocara canis*. Data are presented as the mean values ± SDs of seven animals per time point. Significant differences between the groups are indicated (*p* = 0.0177)

The histopathological evaluation of the livers of uninfected animals showed a normal histological appearance, with the hepatic tissue formed by the lobular vein center surrounded by cords of hepatocytes and sinusoidal capillaries. The hepatocytes presented with a polygonal shape and central spherical nuclei (Fig. 3a). The livers from animals at 7 days p.i. showed regions with moderate focal inflammatory infiltrate, consisting primarily of eosinophils and neutrophils, and small regions were occasionally permeated with necrosis of hepatocytes and polymorphonuclear cell infiltration. Perivascular inflammatory infiltration was also observed (Fig. 3b). The livers of animals at 14 days p.i. showed lesions in the hepatic parenchyma, characterized by large areas with intense inflammatory infiltrates composed of neutrophils and eosinophils, as well as areas with moderate hepatic necrosis associated with polymorphonuclear inflammatory infiltrate. Intensive perivascular inflammatory infiltrates were also present (Fig. 3c and d). The lesion area of the liver was larger due to the duration of the infection (Fig. 4).

**Fig. 5:**
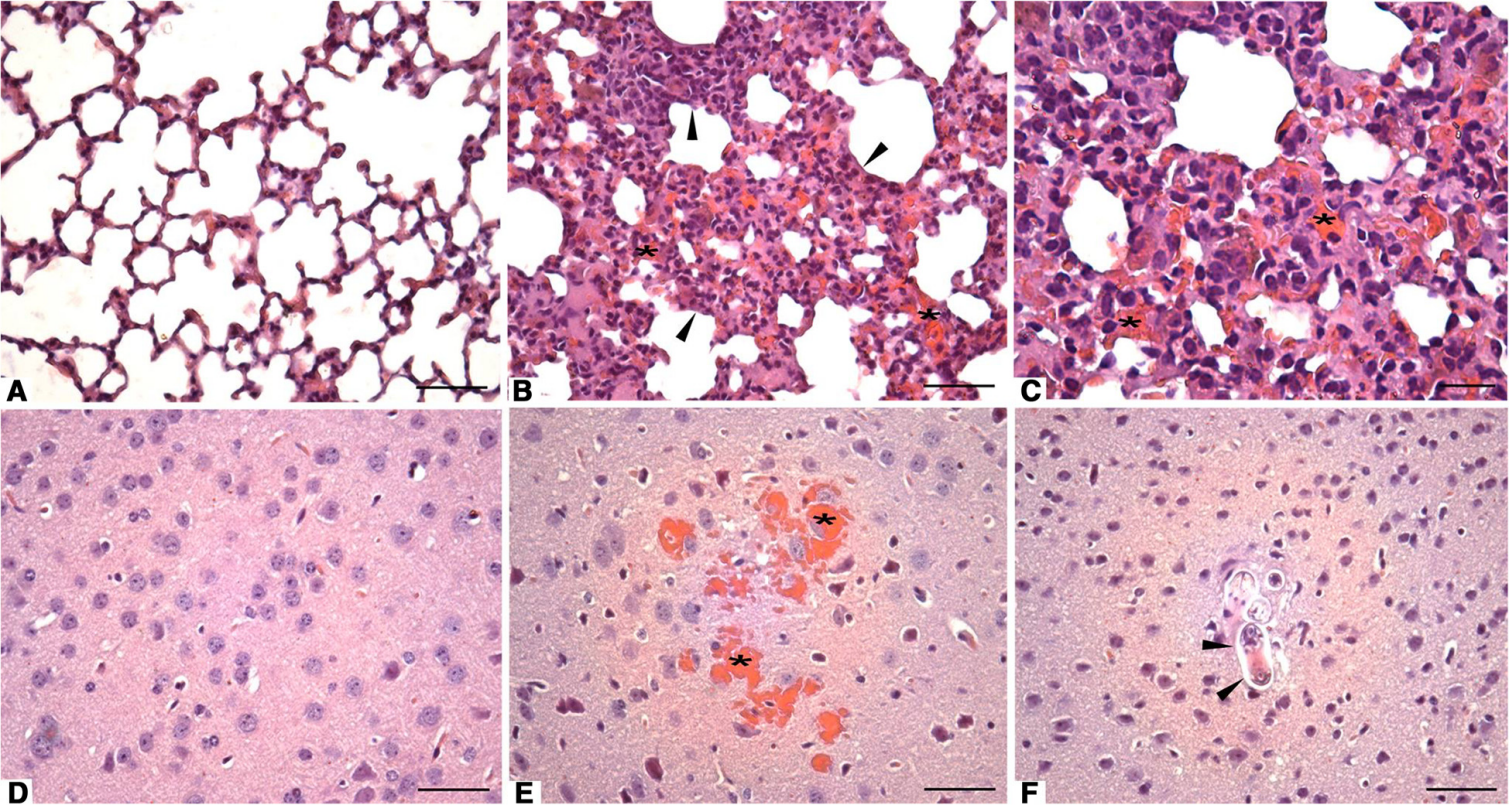
Lung and brain parenchyma of BALB/c mice infected with *Toxocara canis*. **a** Control group: normal lung parenchyma. H&E staining. *Bar* = 50 μm. **b** 14 days post-infection (p.i.): thickening of the septum due to inflammatory infiltrate (*arrowheads*) and hemorrhagic areas (*). H&E. *Bar* = 50 μm. **c** Higher magnification of the previous figure (14 days p.i.) showing inflammatory infiltrates consisting of eosinophils, lymphocytes, macrophages and the presence of hemorrhagic areas (*). H&E. *Bar* = 20 μm. **d** Control group: normal brain parenchyma. H&E. *Bar* = 20 μm. **e** 7 days p.i.: brain with hemorrhagic cavities (*). H&E. *Bar* = 20 μm. **f** 14 days p.i.: presence of larvae in the brain (*arrowheads*). H&E. *Bar* = 20 μm

The histopathological evaluation of the lung parenchyma from uninfected animals showed a normal histological appearance, with aerated alveoli and alveolar and interlobular septa with normal thicknesses (Fig. 5a). The lungs of animals at 7 and 14 days p.i. showed parenchymal lesions characterized by extensive thickening of the septum at the expense of the presence of intense inflammatory infiltrate, composed of eosinophils, lymphocytes, and macrophages, and the presence of hemorrhagic areas (Fig. 5b). Inflammatory infiltrate was found filling the alveolar lumen and along the bronchioles and blood vessels, with the same cellular profile as the septum infiltrate (Fig. 5c). The lesion area of the lungs showed no differences from 7 to 14 days p.i., with lesion areas of 1943 ± 264 and 2242 ± 517 μm^2^, respectively.

The histopathological evaluation of the brains of uninfected animals showed a normal histological appearance (Fig. 5d). Brains from infected animals at 7 and 14 days p.i. presented variable-sized cavities partially filled with blood (hemorrhagic cavities) (Fig. 5e), and scattered larvae were visualized in the cerebrum and brainstem (Fig. 5f). The lesion areas of the brain showed no difference from 7 to 14 days p.i., with hemorrhagic areas of 1634 ± 1088 and 859 ± 492 μm^2^, respectively.

### Hematological profile of *T. canis*-infected BALB/c mice

The hematological profile was evaluated at all of the time points of infection (Table 1). The most important findings demonstrated a progressive increase in neutrophils, which peaked on the 7th day of infection and returned to baseline levels at the 14th day p.i. Moreover, peripheral eosinophilia was also evident after 7 days of infection, persisting to up to 14 days p.i. The monocyte counts showed no variation during the experiments.

**Table 1:** Peripheral blood cells of *Toxocara canis*-infected BALB/c mice

	Days post-infection					
	0	1	3	5	7	14
WBC (× 10^3^/μL)	4.6 ± 1.4	2.6 ± 0.3	4.0 ± 0.4	5.2 ± 1.5	6.8 ± 2.1^b,c^	6.3 ± 1.5^b,c^
Lymphocyte (× 10^3^/μL)	3.45 ± 1.11 (77–87 %)	1.40 ± 0.15 (47–61 %)	2.50 ± 0.18 (58–68 %)	3.11 ± 0.88 (57–63 %)	3.93 ± 1.23 (51–67 %)	3.54 ± 1.18 (47–63 %)
Monocyte (× 10^3^/μL)	0.01 ± 0.01 (0–1 %)	0.03 ± 0.03 (0–3 %)	0.02 ± 0.02 (0–1 %)	0.01 ± 0.02 (0–2 %)	0.07 ± 0.06 (0–2 %)	0.03 ± 0.04 (0–2 %)
Neutrophil (× 10^3^/μL)	1.10 ± 0.42 (11–23 %)	1.15 ± 0.31 (35–51 %)	1.41 ± 0.34 (30–40 %)	1.86 ± 0.66 (32–38 %)	2.25 ± 1.12^b,c,f^ (23–41 %)	1.00 ± 0.32 (13–23 %)
Eosinophil (× 10^3^/μL)	0.03 ± 0.02 (0–2 %)	0.06 ± 0.03 (1–5 %)	0.07 ± 0.03 (1–3 %)	0.21 ± 0.07 (3–5 %)	0.48 ± 0.18^a,b,c,f^ (5–13 %)	1.68 ± 0.48^a,b,c,d,e^ (21–33 %)
RBC (× 10^6^/μL)	7.0 ± 2.0	9.3 ± 0.7	9.2 ± 1.0	5.8 ± 1.0*	8.1 ± 1.3	8.0 ± 0.7
Hemoglobin (g/dL)	16.7 ± 1.2	17.0 ± 1.4	17.6 ± 1.6	13.3 ± 1.3*	15.8 ± 1.4	17.0 ± 1.3
Platelet (× 10^3^/μL)	722 ± 35	725 ± 58	598 ± 29	766 ± 57	758 ± 31	725 ± 47

*WBC* white blood cells, *RBC* red blood cells. *n* = six animals per time point of infection. Data are presented as mean ± SDs and relative values. For leukocytes population, significant differences (*p* ≤ 0.05) related to 0, 1, 3, 5, 7 and 14 dpi are represented by a, b, c, d, e and f, respectively. For red blood cells, significant differences (*p* ≤ 0.05) are represented by * in relation to control group

Analysis of the red blood cell compartments demonstrated a significant reduction in total erythrocytes and hemoglobin levels on the 5th day of infection, compared to the other groups. No differences were observed in platelet counts.

### Plasmatic cytokine profile during early *T. canis* infection

To characterize the systemic immune response during early *T. canis* infection, the plasma levels of T_H_1, T_H_2, T_H_17 and regulatory cytokines were measured at different time points of infection.

**Fig. 6:**
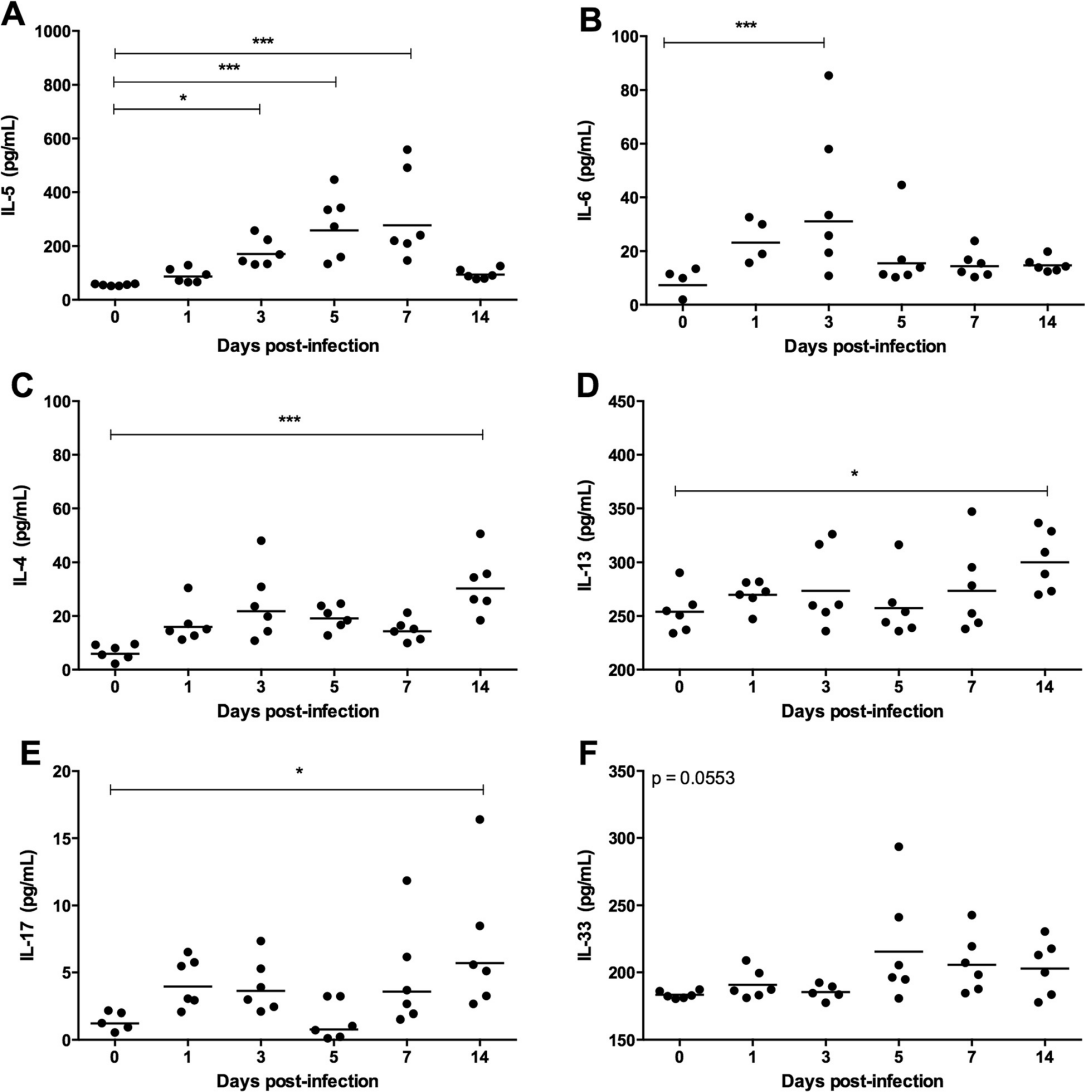
Plasmatic IL-4, IL-5, IL-6, IL-13, IL-17 and IL-33 cytokine levels in *Toxocara canis-*infected BALB/c mice. **a** Plasmatic IL-5 level per days post-infection. **b** Plasmatic IL-6 level per days post-infection. **c** Plasmatic IL-4 level per days post-infection. **d** Plasmatic IL-13 level per days post-infection. **e** Plasmatic IL-17 level per days post-infection. **f** Plasmatic IL-33 level per days post-infection. Each group consisted of six animals per time point of infection. Data are presented as geometric means. Significant differences between the groups are indicated by *asterisks* (*p < 0.05, **p < 0.01 and ***p < 0.001)

Notably, *T. canis* parasites induced a mix of innate inflammatory/T_H_17 andT_H_2 responses during early larval migration, with a predominance of the latter. The T_H_2 response was evidenced by significant increases in IL-4, IL-5, IL-13 and IL-33 levels. Additionally, increasing levels of IL-6 and IL-17 were also observed during the early course of infection (Fig. 6). In contrast, no differences were observed for systemic TNF-α or IFN-γ production (data not shown).

The results related to IL-5 revealed an early, significant and progressive increase in this cytokine from the 3rd day of infection until the 7th day p.i., which then decreased to baseline values (Fig. 6a). This early increase was also seen for IL-6 secretion on the 3rd day of infection (Fig. 6b). Interestingly, the IL-4, IL-13 and IL-17 secretion patterns were similar and were characterized by a significant increase after 14 days p.i. (Fig. 6c, d and e). Finally, IL-33 revealed slightly elevated concentrations in plasma samples from infected animals from the 5th day onward; however, the increase was not statistically significant compared with uninfected animals (Fig. 6f). No significant increase in IL-10 secretion was observed during the early phase of infection with *T. canis* (data not shown).

## Discussion

With the aim of studying the manifestations of early *T. canis* infection in mice, we evaluated the kinetics of larval recovery in different organs, as well as immunopathological aspects of the affected tissues and systemic immune responses during the first 2 weeks of infection.

Studies of the effects of migrating *Toxocara* larvae in paratenic animal models are justified by a certain similarity to human toxocariasis, and these models can elucidate unknown mechanisms of host-parasite interaction [13]. In the current work, the use of female BALB/c mice was justified due to the animals’ susceptibility to *T. canis* larvae and the resulting higher parasite loads than in other mouse strains, particularly in the brain, making these mice a suitable model for studies of visceral toxocariasis and neurotoxocariasis [22].

In the environment, unembryonated *T. canis* eggs from the feces of dogs and cats will develop into infective eggs under optimal temperature and humidity within 9–15 days (see Schnieder et al. [23] for a review). In cold, temperate regions, *T. canis* eggs in the soil were reported to become infective after 4–6 weeks [24]. Here, in vitro conditions, as previously described for *Ascaris* spp. egg embryonation [18], were employed to obtain the infective stages of the eggs after 4–6 weeks.

Concerning the migratory route of *T. canis* larvae, a previous review by Othman [25] showed that there have been controversies about the migratory route and accumulation of larvae in different organs during the acute and chronic phases. The descriptions of findings and recovery of motile larvae from the different tissues of infected mice have led to the conclusion that larval migration is a continuous process, most probably not consisting of just a simple one-way route from the intestinal wall through lungs and to the brain [25]. However, in the present study, we were able to demonstrate a clear concentration of migrating larvae in the liver (day 1 p.i.), lungs (day 3 p.i.) and brain (from day 5 p.i. onwards), at least during the initial phase of acute infection. These results show that there is a clear route of migration by the majority of *T. canis* larvae in BALB/c mice and that the neurological sequelae which are evident as soon as 5 days p.i. follow because some larvae reach the brain as early as days 3–5. Nevertheless, our results do not exclude the possibility that some larvae venture into other organs and tissue sites such as muscles which were not studied in the current work.

A similar time frame of brain infection was observed when BALB/c mice were infected with limited doses of embryonated eggs, and the number of recovered larvae from brain tissue was shown to be proportional to the number of inoculated infective stages [26]. These authors explained this phenomenon mainly by the incapacity of the liver to retain the larvae, thus allowing larva migration to the brain. The tendency toward accumulating larvae in the brain might occur because the parasites induce behavioral changes in the paratenic host and increase their likelihood of being predated by the definitive host (reviewed by Schnieder et al. [23]), or it might occur as part of its immune evasion strategy to seek immune-privileged infection sites (see Othman [25] for review).

A histopathological study of *T. canis*-infected mice over 67 weeks revealed that granulomatous inflammatory lesions were commonly observed in the liver, lungs, and musculature from 1 week p.i. onward, but these lesions were rarely seen in the brain [27]. Trapped larvae, however, were found only in histological sections from 12 weeks p.i. or later [27]. Another histopathological study of liver alterations in infected Swiss mice revealed inflammatory reactions in the forms of plasma cells, neutrophils, eosinophils, and aggregates of lymphocytes around the central veins and in the liver parenchyma at 14 days p.i. [28]. In our experiments, the main cell types at the inflammation sites in the liver consisted of eosinophils, neutrophils and polymorphonuclear cells, without a significant contribution of lymphocytes at 7 or 14 days p.i. Interestingly, the high neutrophil counts followed the migration of larvae through the organs and tissues, likely acting as the first line of defense against *T. canis* larvae, as proposed for protozoan parasites [29]. Their roles during toxocariasis in paratenic hosts should be investigated in further studies.

Analysis of pulmonary histopathology indicated eosinophils, lymphocytes and macrophages as the main components of inflammatory infiltrates, both at 7 and at 14 days p.i. These results and other findings in the lungs corroborated the previous findings published by Pinelli et al. [15, 17] of pathological changes in the lungs 7 to 60 days after *T. canis* infections. Additionally, our results regarding brain lesions were in agreement with those of Othman et al. [30], who showed that, in Swiss mice infected with *T. canis*, motile larvae were more abundant in the cerebrum, without being trapped by an inflammatory reaction, thus causing vascular congestion and hemorrhages in the brain. The cerebrum as the preferential location of larvae in the brain was also reported by Janecek et al. [31].

To accompany the findings of larval recovery, hematology and histopathology, we measured the secretion of cytokines in peripheral blood samples during early *T. canis* infection. In accordance with the elevated numbers of neutrophils and eosinophils in the peripheral blood and histological sections, we detected prominent increases in T_H_2 cytokines, such as IL-4, IL-5, IL-13 and IL-33, during the acute phase of infection, particularly from the 3rd day p.i. onward. However, the maximum T_H_2 response and the recruitment of innate effector cells seemed to lag behind the rapid migration of larvae through the tissues of the liver and lungs, and during this early phase, no granuloma formation or trapping of larvae was observed in tissues. Based on unspecific and parasite-specific antibody responses, polarization toward a T_H_2 immune response at later time points of experimental *Toxocara* infection has already been reported by others [17, 27]. Notably, the quantification of IL-4 and IL-5 levels by PCR in the lungs at 7 and 14 days p.i. [17] demonstrated similar cytokine profiles to those observed in our study from the plasma samples of infected animals. While these authors found elevated levels of IL-10 in the lungs at 7 and 14 days p.i., we did not find elevated IL-10 concentrations in peripheral blood throughout our experiments, indicating that the production of this regulatory cytokine occurred only at the site of infection.

Takamoto et al. [32] showed that *Toxocara*-infected mice showed a biphasic rise in eosinophilia during infection. These authors were able to show, in CD4-deficient mice, that the first increase, at 10 days p.i., was part of the innate immune response, whereas the second peak in eosinophilia, at 3 weeks p.i., was dependent on CD4 T cells and was a part of the adaptive immune response. We also measured increases in eosinophil counts at 7 and 14 days p.i. as part of the innate immune response. Compared with eosinophil counts, the initial increase in neutrophil counts in the peripheral blood until 7 days p.i. was more prominent, and as such, these cells seemed to play an important role during innate anti-parasite responses. In addition to the decrease in neutrophil counts on day 14, similar responses in the hematological profile were reported by Pecinali et al. [16].

Apart from the T_H_2 cytokines, IL-6 and IL-17 were detected in peripheral blood during the first three days of infection. Whereas IL-6 decreased to baseline levels on subsequent days, elevated concentrations of IL-17 in the peripheral blood were still detected on days 7 and 14 p.i. IL-6 is considered one of the major pro-inflammatory cytokines, and it acts on a variety of cells, including immune-competent cells and hematopoietic cells, to cause proliferation and differentiation. It is produced by various cell types, such as T cells, B cells, monocytes, macrophages, dendritic cells, fibroblasts, endothelial cells, and glial cells, and it regulates the immune response and inflammation [33]. Furthermore, in conjunction with TGF-β, IL-6 links innate immune responses to T cell effector mechanisms and promotes the development of T_H_17 cells [34]. IL-6 plays a key role in the development of the CD4 T_H_17 cell line, and both IL-6 and IL-17, if upregulated, can lead to chronic inflammatory processes and disease [35].

Most recently, it was shown in *Nippostrongylus brasiliensis*-infected BALB/c mice that host chitinase-like proteins (CLPs) induced neutrophil activation and, together with IL-17A produced by activated γδ T cells, contributed to larval elimination at the price of enhanced lung damage during larval migration [36]. To limit acute tissue damage, a switch to adaptive T_H_2 responses was shown from the 4th day p.i. for the same parasite [37]. In our model, we found elevated T_H_2 cytokines in the peripheral blood from the 3rd day p.i. onward, without any contribution of IFN-γ.

Therefore, similar mechanisms and timing of immune responses might occur during infection with *T. canis* as well. However, even with a driven and robust humoral and cellular immune response, *T. canis* may survive for extended periods, suggesting that this nematode develop efficient strategies to escape the immune destruction. In fact, several mechanisms of immune evasion were reported for *T. canis* and some excretory/secretory products of this parasite, such as TES-32 [38], TES-70 [39], and CTL-1 (C-type lectin 1) [40] were already implicated to regulate the host immunity to *T. canis* infection. In this context, the contribution of these products and its importance into the parasite-host interaction should be issues of future research in early experimental toxocariasis.

## Conclusions

In conclusion, we presented new insights into pro-inflammatory responses, innate effector cell activation and polarized T_H_2 immune responses during early *T. canis* infection in experimentally infected BALB/c mice. The results facilitated a better understanding of immunopathological events that also might occur during human toxocariasis, thus contributing to future strategies of diagnosis and control.
